# Supplementary material for: A survey on text classification: Practical perspectives on the Italian language
Source: PLoS One. 2022 Jul 6;17(7):e0270904. doi: 10.1371/journal.pone.0270904 (PMC9258888; doi:10.1371/journal.pone.0270904)

Datasets statistics

In this supplemental section, we provide integrative information on the composition of the datasets utilized in our work. Analysis and discussions are included in the main article.

Number of labels per article

Fig 1. N. of labels distribution for EnWiki-100

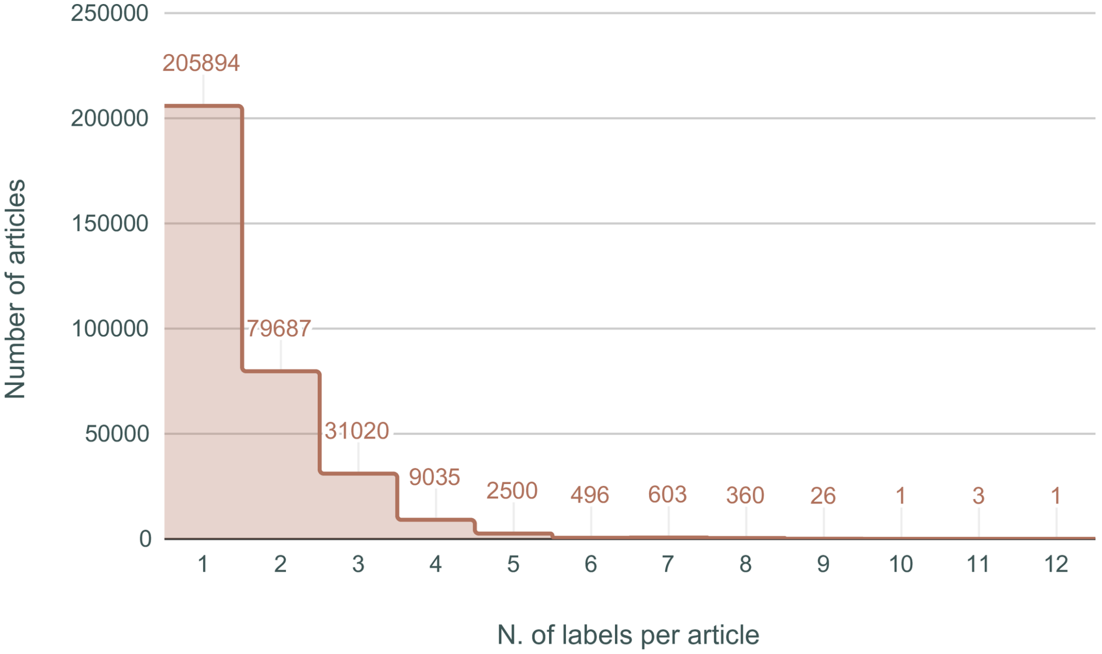

Fig 2. N. of labels distribution for FrWiki-100

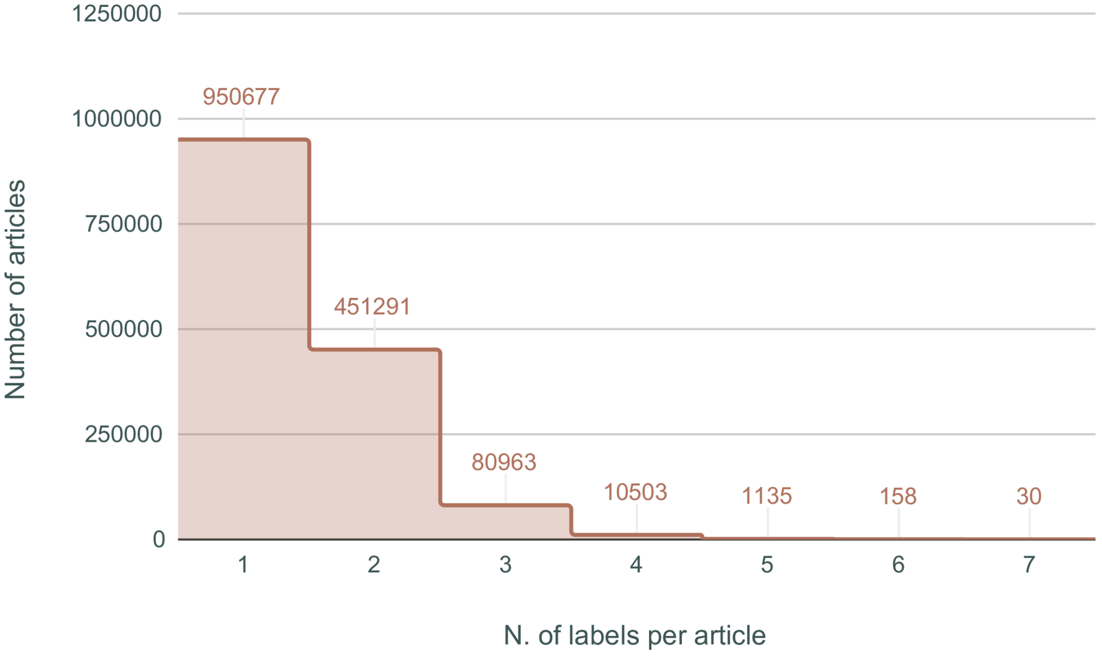

Fig 3. N. of labels distribution for RCV1en

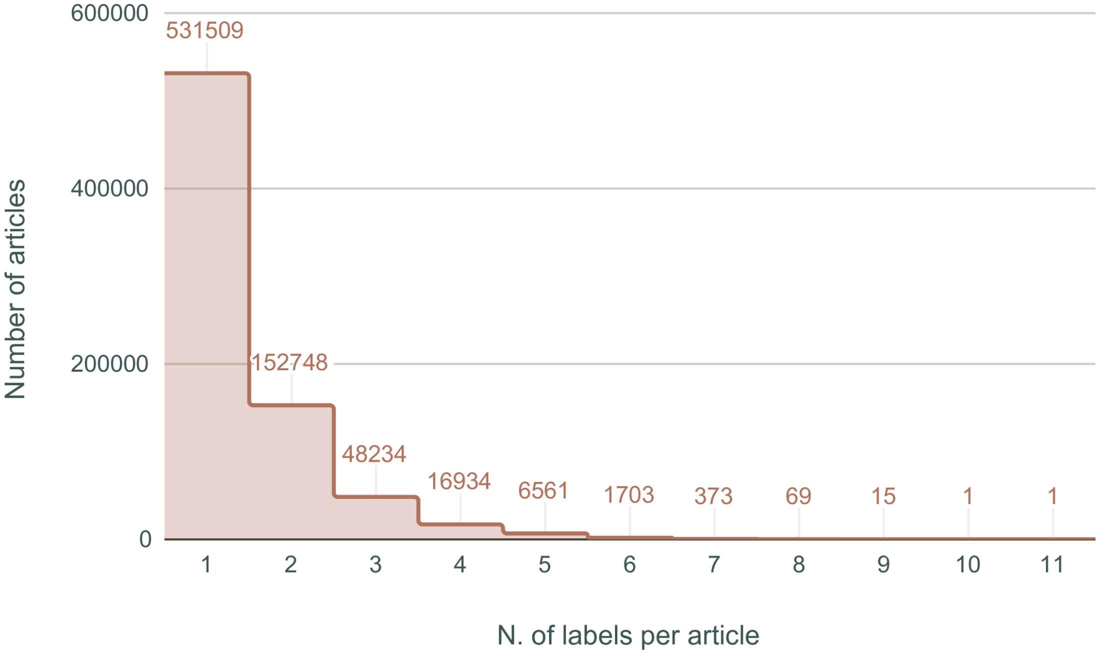

Fig 4. N. of labels distribution for RCV2fr

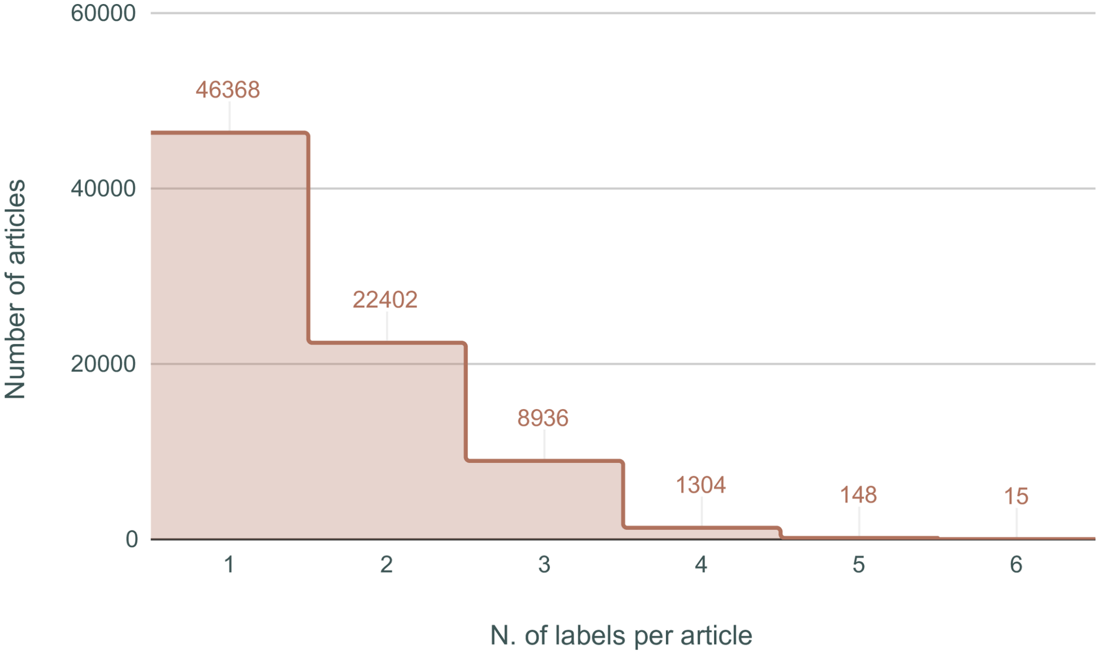

Topic distribution

Fig 5. EnWiki-100 topic distribution (logarithmic scale)

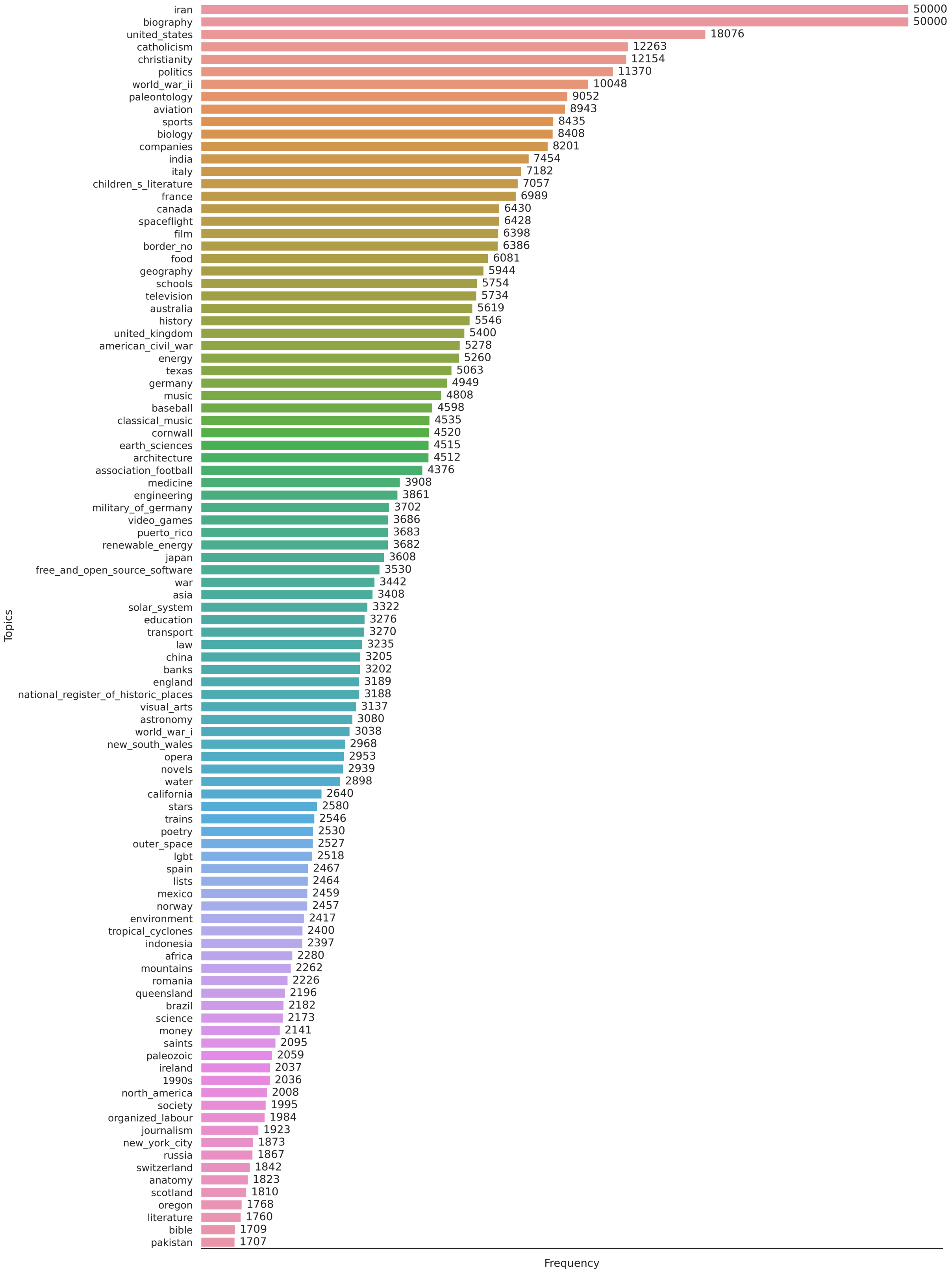

Fig 6. FrWiki-100 topic distribution (logarithmic scale)

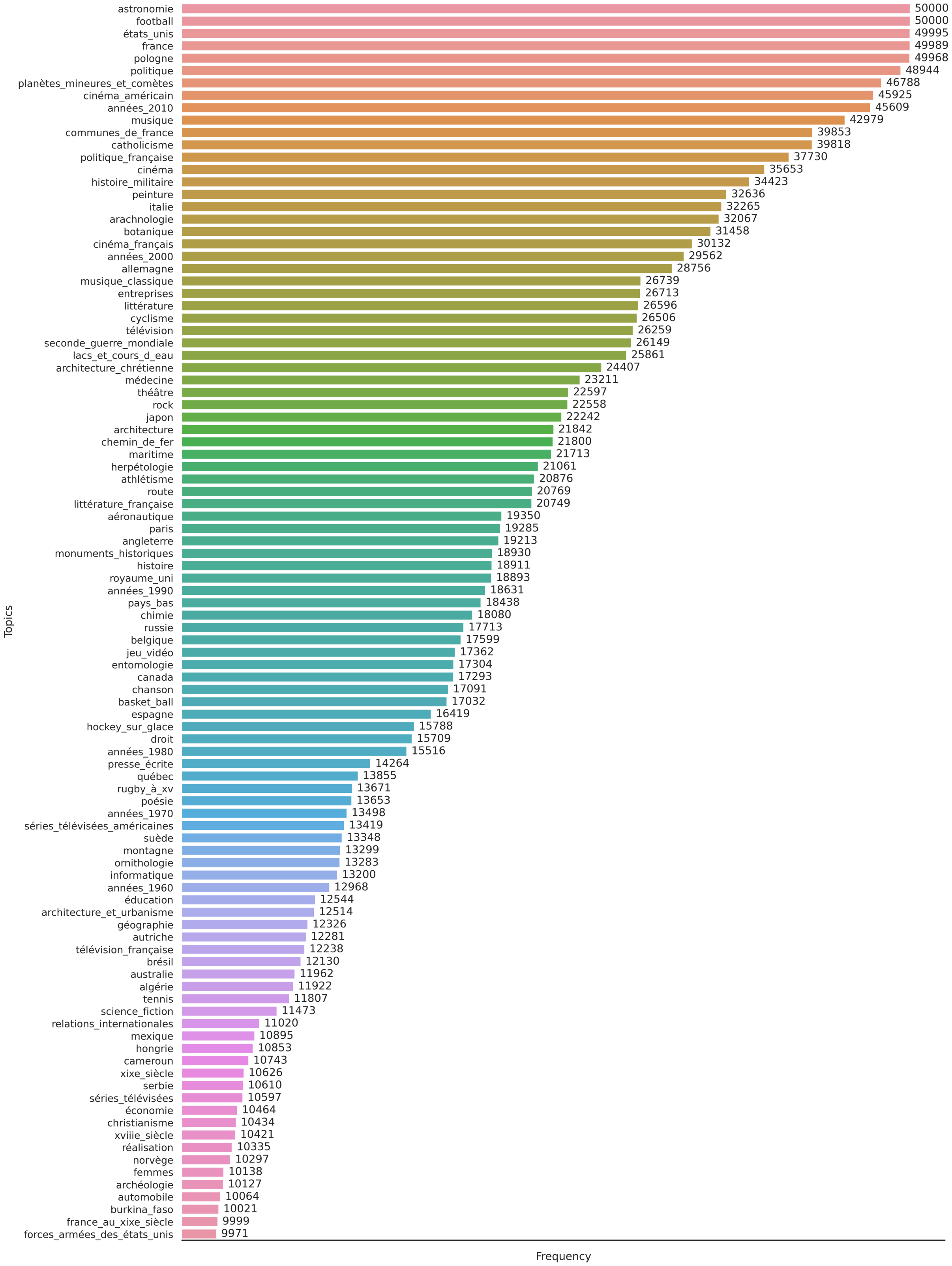

Fig 7. ItWiki-100 topic distribution (logarithmic scale)

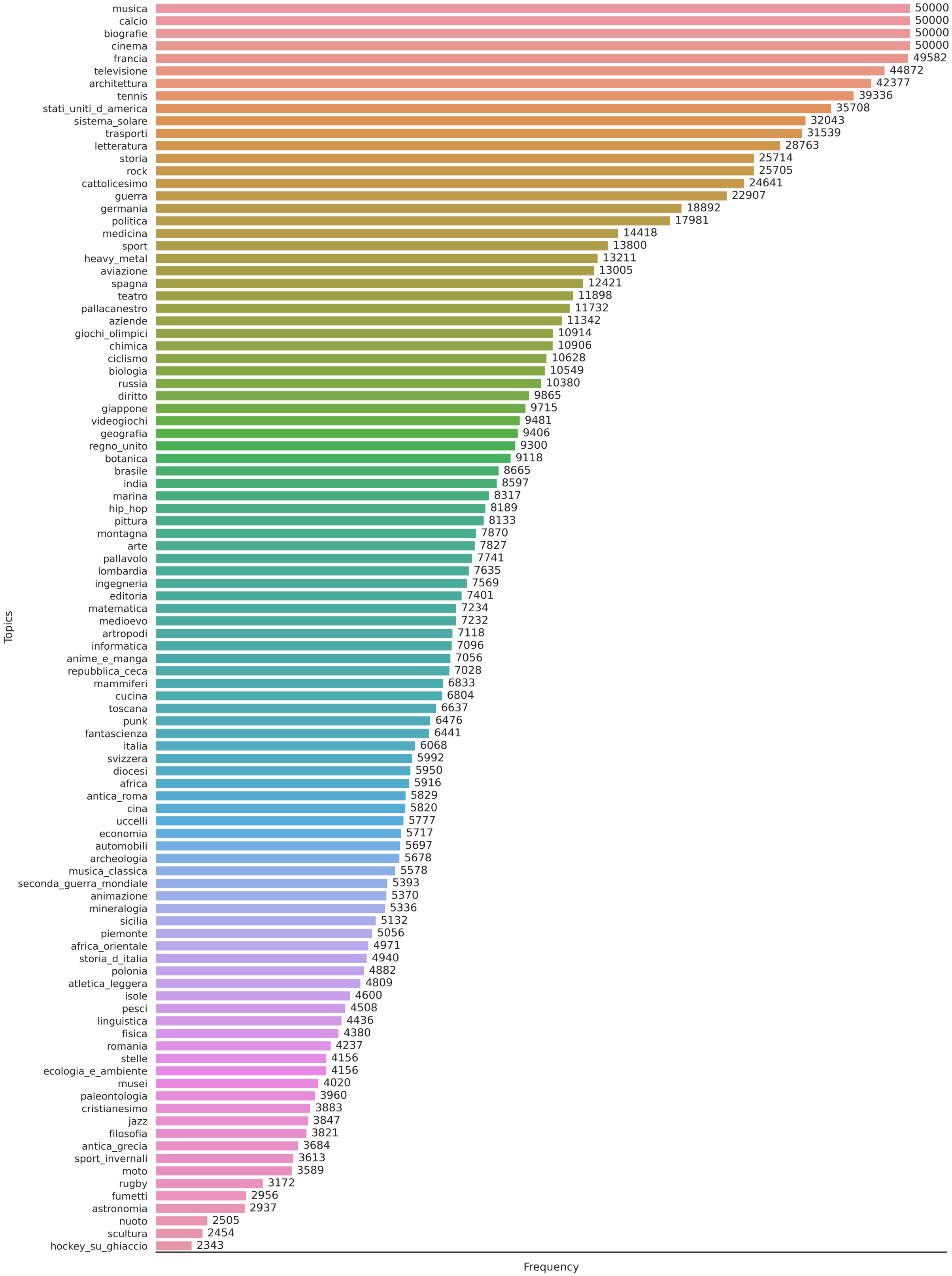

Fig 8. RCV1en topic distribution (logarithmic scale)

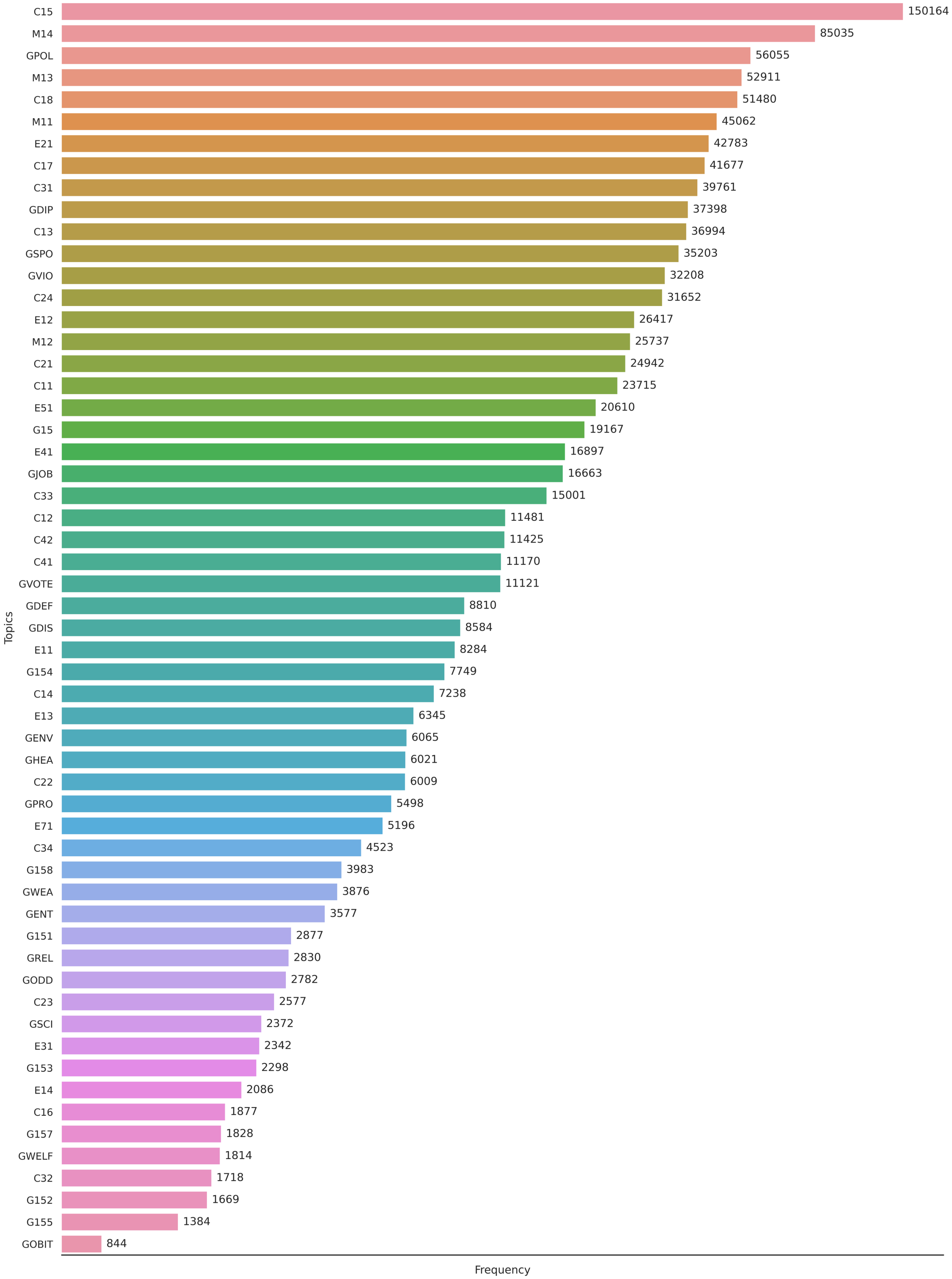

Fig 9. RCV2fr topic distribution (logarithmic scale)

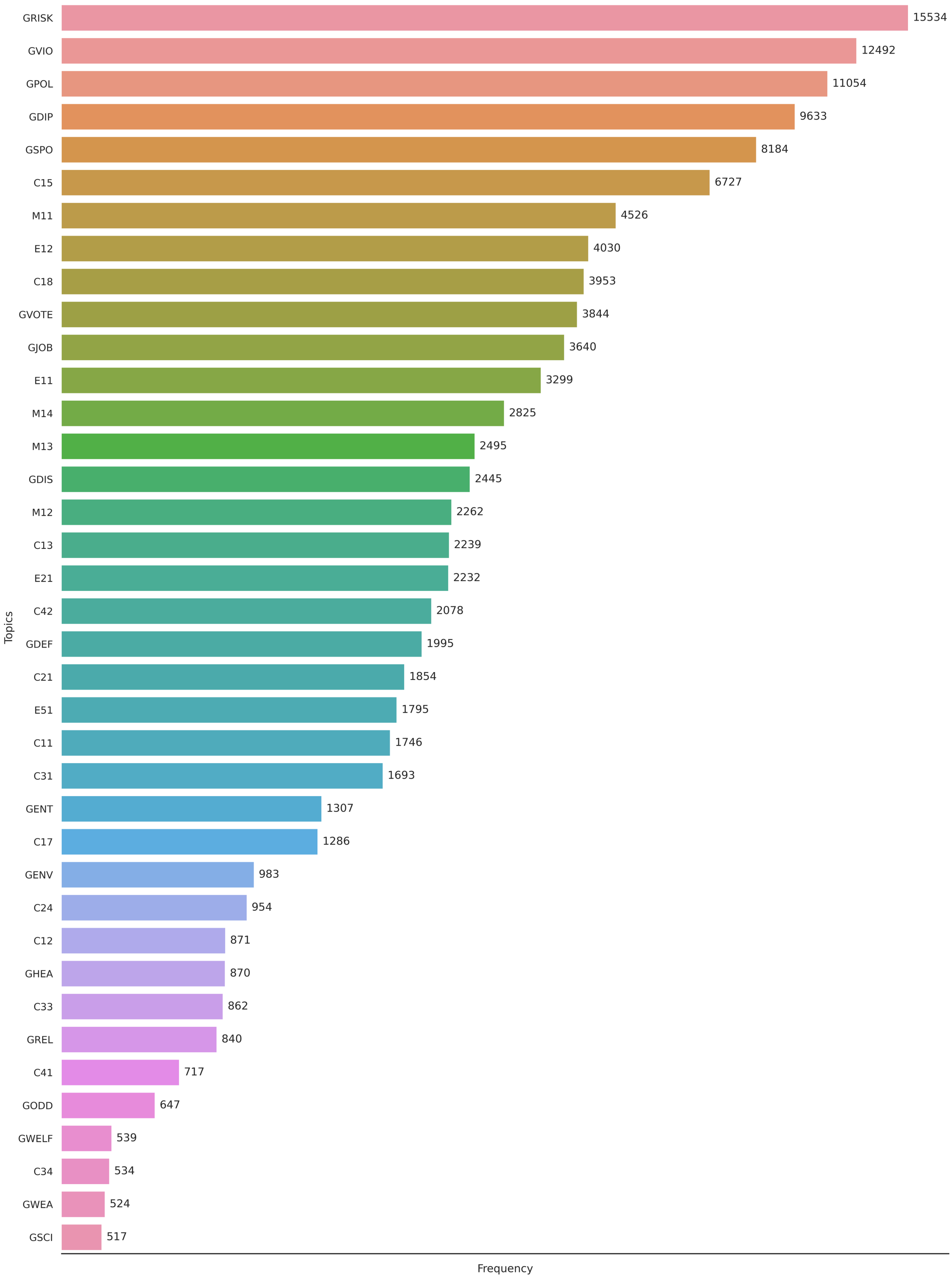

Fig 10. RCV2it topic distribution (logarithmic scale)

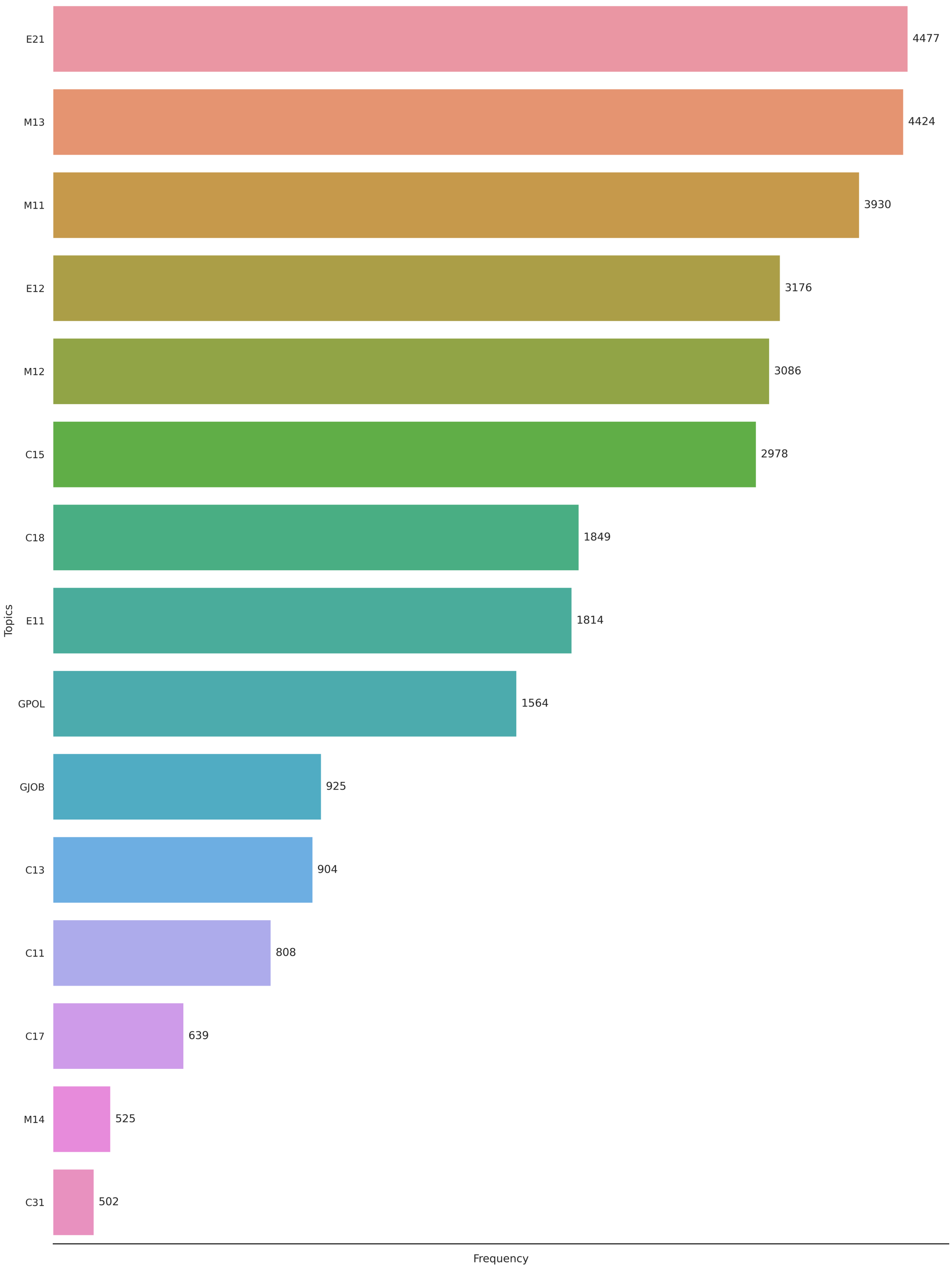

Supplement: S1 Datasets — Histograms with statistics about the RCV1/2 and Wiki datasets. (PDF) [file pone.0270904.s001.pdf]
