# Supplementary material for: A survey on text classification: Practical perspectives on the Italian language
Source: PLoS One. 2022 Jul 6;17(7):e0270904. doi: 10.1371/journal.pone.0270904 (PMC9258888; doi:10.1371/journal.pone.0270904)
Supplement: S1 Appendix — Hyper-parameters used for training and relevant preprocessing operations. (PDF) [file pone.0270904.s002.pdf]

Training and testing procedure

In this supplemental section, we provide details on the choice of models, how they were trained and other related considerations.

Choice of models

The choice of models was based on an empirical evaluation of candidates that could be competitive, while also keeping in mind the technical constraints of our particular setting.

Classic methods As far as traditional methods are concerned, we considered multiple seminal approaches, such as multilabel adaptations of Linear Regression, k-Nearest Neighbors and Random forests. However, these methods did not yield interesting nor notable results, and did not scale well with the problem in question. Therefore, we reported results for a Naïve Bayes multinomial adaptation [1], which was notable in terms of speed, and a linear kernel-based SVM [2], which instead provided excellent results.

Neural approaches As far as neural approaches go, our aim was to test different pre-trained word embeddings as well as neural approaches used before the "Transformer era". The FastText [3] library provides both an excellent source of static embeddings, as well as an out-of-the-box classifier (based on shallow MLPs). XML-CNN [4] was chosen as an approach specific to multilabel classification and a representative of CNN-based architectures. We initially tested a few different recurrent architectures, but the simple Bidirectional LSTM with max pooling over time (similar to [5, 6]) provided the best results. Other methods tested include the Deep Pyramid Convolutional Neural Network [7] and the Recurrent Convolutional Neural Network [8], whose results were, on average, the same or worse than the models proposed. The implementations we tested are nonetheless still available at `https://gitlab.com/distration/dsi-nlp-publib`.

In terms of embeddings, FastText [3] and GloVe [9] embeddings were the most reliable source of multilingual static embeddings we could find. Table 1 specifies the embeddings utilized by all methods for the best results obtained.

Deep language models Finally, our choice of BERT and XLM-R [10, 11] as contextualized language model was imposed by the pre-trained models available at the time of testing. In the first case, we had to choose a comparable architecture for which we could reliably find pre-trained models in all three languages. As reported in the main article, we found less than 10 non-finetuned Italian language models. We made an empirical evaluation of the corpora they were trained on in deciding which model use, though they were all based on either BERT, RoBERTa or GPT-2. As far as multilingual models go, we tested both multilingual BERT and XLM-R, and chose the latter because of its higher performance.

While more recent and better performing LMs exist (such as XLNet [12]), there are no pre-trained resources available for the Italian (nor French) language. Without the computational resources to perform these procedures (which are exceptionally high), these models cannot be tested.

Training details

Tables 1 and 2 highlight the most prominent information about the training procedure for the algorithms utilized in our experiments. Traditional methods were trained until convergence. Non-transformer based neural networks were trained with early stopping (2 consecutive epochs without

improvement), while BERT-based networks were fine-tuned for 2 to 4 epochs, following the suggestions by the original authors. During training of all neural networks, we utilized AdamW [13] as optimizer, and binary cross entropy as the objective function (as is standard for multilabel classification problems).

Classic methods We train Naïve Bayes for multinomial distributions and SVM classifiers with linear kernel, utilizing a one-vs-rest strategy to suit them for multilabel classification. We perform a grid search of hyperparameters and apply a 10-fold cross validation procedure. The input of these classifiers are TF-IDF vectors obtained on the respective corpora after it has been cleaned of stopwords and tokenized at word level; the maximum number of features is limited to the 30,000 words with highest weight.

Neural approaches We utilized FastText's out-of-the-box classifier as provided in its official Python library. For classification, FastText extracts n-gram features that are averaged and then fed to a linear classifier with softmax output [14]. Since FastText provides an embedded procedure for hyperparameters tuning, we run this for 30 minutes on the validation set to obtain a set of optimized parameters, and we then train the model with them. The only hyperparameter manually chosen is the loss function, which we set to a one-vs-all policy, as suggested in the documentation for a multilabel use case. We train XML-CNN with the hyperparameters as recommended by its authors and only calibrate the learning rate by choosing the one that achieves best results during validation, with batch size set to 64. The inputs to the network are documents where words are represented by pre-trained embeddings. A similar approach was taken for the BiLSTM. The batch size was also set to 64. For both this model and XML-CNN, we allowed the fine-tuning procedure to affect the pre-trained embeddings, as it achieved better results.

Deep language models Lastly, as representatives for Transformer pre-trained language models, we make use of weights made available on the Hugging Face library [15]. The models are adapted to our classification tasks by adding a linear layer on top of the base architecture. We fine-tune them for a maximum of 4 epochs with early stopping set on macro F1-score. While hyperparameters are often task-specific, we follow the advice of Devlin et al. [10], which suggest fine-tuning BERT on downstream tasks for 2 to 4 epochs with a batch size of 16 to 32 and learning rate of $2 \cdot 10^{-5}$ to $5 \cdot 10^{-5}$ for optimal results. Due to limitations in our computational resources we trained all models with batch size set to 8 or 10. Therefore, we lowered the learning rate to $1.5 \cdot 10^{-5}$ for all runs.

Table 1: Neural Network hyper-parameters.

Model	Embedding size	Batch Size	Epochs	Optimizer	Learning rate	Weight Decay
BiLSTM	512	64	4 to 9	Adam	$5,0 \cdot 10^{-4}$	-
XLM-CNN	512	64	10 to 20	Adam	$2,0 \cdot 10^{-4}$	-
BERT	768	8 to 10	2 to 4	AdamW	$1,5 \cdot 10^{-5}$	$1,0 \cdot 10^{-2}$
XLM-R	768	4	2 to 4	AdamW	$1,5 \cdot 10^{-5}$	$1,0 \cdot 10^{-2}$

Table 2: Technical specifications.

Model	Input	Preprocessing	Additional info
Naïve Bayes	TF-IDF vectors	Stopword removal, Word-level tokenization, Top 30,000 features	One-vs-All, Multinomial, 10-fold CV
SVM	TF-IDF vectors	Stopword removal, Word-level tokenization, Top 30,000 features	One-vs-All, Linear kernel, 10-fold CV
FastText	FastText embeddings	N/A (internal)	One-vs-All, 30 min tuning
BiLSTM	GloVe embeddings	Stopword removal, Word-level tokenization	Fine-tune embeddings
XML-CNN	FastText embeddings	Stopword removal, Word-level tokenization	Fine-tune embeddings
BERT	Tokenized text	WordPiece tokenizer	Fine-tune embeddings
XLM-R	Tokenized text	SentencePiece tokenizer	Fine-tune embeddings

References

[1] Xu S, Li Y, Zheng W. Bayesian Multinomial Naïve Bayes Classifier to Text Classification; 2017. p. 347–352.

[2] Boser BE, Guyon IM, Vapnik VN. A Training Algorithm for Optimal Margin Classifiers. In: Proceedings of the Fifth Annual Workshop on Computational Learning Theory. Association for Computing Machinery; 1992. p. 144–152.

[3] Bojanowski P, Grave E, Joulin A, Mikolov T. Enriching Word Vectors with Subword Information. Transactions of the Association for Computational Linguistics. 2017;5:135–146. doi:10.1162/tacl_a_00051.

[4] Liu J, Chang WC, Wu Y, Yang Y. Deep Learning for Extreme Multi-Label Text Classification. In: Proceedings of the 40th International ACM SIGIR Conference on Research and Development in Information Retrieval. SIGIR '17. Association for Computing Machinery; 2017. p. 115–124.

[5] Zhou P, Qi Z, Zheng S, Xu J, Bao H, Xu B. Text Classification Improved by Integrating Bidirectional LSTM with Two-dimensional Max Pooling. In: Proceedings of COLING 2016, the 26th International Conference on Computational Linguistics: Technical Papers. The COLING 2016 Organizing Committee; 2016. p. 3485–3495.

[6] Kao CC, Sun M, Wang W, Wang C. A Comparison of Pooling Methods on LSTM Models for Rare Acoustic Event Classification. CoRR. 2020;.

[7] Johnson R, Zhang T. Deep Pyramid Convolutional Neural Networks for Text Categorization. In: Proceedings of the 55th Annual Meeting of the Association for Computational Linguistics (Volume 1: Long Papers). Vancouver, Canada: Association for Computational Linguistics; 2017. p. 562–570. Available from: https://aclanthology.org/P17-1052.

[8] Lai S, Xu L, Liu K, Zhao J. Recurrent Convolutional Neural Networks for Text Classification. In: Proceedings of the Twenty-Ninth AAAI Conference on Artificial Intelligence. AAAI'15. AAAI Press; 2015. p. 2267–2273.

[9] Pennington J, Socher R, Manning C. GloVe: Global Vectors for Word Representation. In: Proceedings of the 2014 Conference on Empirical Methods in Natural Language Processing (EMNLP). Association for Computational Linguistics; 2014. p. 1532–1543.

[10] Devlin J, Chang MW, Lee K, Toutanova K. BERT: Pre-training of Deep Bidirectional Transformers for Language Understanding. In: Proceedings of the 2019 Conference of the North American Chapter of the Association for Computational Linguistics: Human Language Technologies, Volume 1 (Long and Short Papers). Association for Computational Linguistics; 2019. p. 4171–4186.

[11] Conneau A, Khandelwal K, Goyal N, Chaudhary V, Wenzek G, Guzmán F, et al. Unsupervised Cross-lingual Representation Learning at Scale. In: Proceedings of the 58th Annual Meeting of the Association for Computational Linguistics. Association for Computational Linguistics; 2020. p. 8440–8451.

[12] Yang Z, Dai Z, Yang Y, Carbonell J, Salakhutdinov R, Le QV. XLNet: Generalized Autoregressive Pretraining for Language Understanding. In: Proceedings of the 33rd International Conference on Neural Information Processing Systems. Curran Associates Inc.; 2019.

[13] Loshchilov I, Hutter F. Fixing Weight Decay Regularization in Adam. CoRR. 2017;abs/1711.05101.

[14] Joulin A, Grave E, Bojanowski P, Mikolov T. Bag of Tricks for Efficient Text Classification. In: Proceedings of the 15th Conference of the European Chapter of the Association for Computational Linguistics: Volume 2, Short Papers. Association for Computational Linguistics; 2017. p. 427–431.

[15] Wolf T, Debut L, Sanh V, Chaumond J, Delangue C, Moi A, et al. Transformers: State-of-the-Art Natural Language Processing. In: Proceedings of the 2020 Conference on Empirical Methods in Natural Language Processing: System Demonstrations. Association for Computational Linguistics; 2020. p. 38–45.
