# Supplementary material for: A survey on text classification: Practical perspectives on the Italian language
Source: PLoS One. 2022 Jul 6;17(7):e0270904. doi: 10.1371/journal.pone.0270904 (PMC9258888; doi:10.1371/journal.pone.0270904)
Supplement: Attachment — Submitted filename: rebuttal.pdf [file pone.0270904.s003.pdf]

Response to Reviewers

Dear *PLOS ONE* Editors and Reviewers,

We would like to thank the reviewers for their feedback. We have carefully considered your requests and suggestions, and addressed each and every one of them.

First off, in response to the editor's request, we have reviewed PLOS ONE's style requirements, and have done our best to update the manuscript to precisely meet them.

In response to prompt (a) and (b), we note that the Reuters Corpora utilized in our experiments is owned by NIST, who acts as its sole distributor. In particular, the researchers are asked to sign an agreement by which "the display, reproduction, transmission, distribution or publication of the information is prohibited" and "summaries, analyses and interpretations of the linguistic properties of the information may be derived and published, provided it is not possible to reconstruct the information from these summaries". These restrictions are documented at `https://trec.nist.gov/data/reuters/reuters.html`. On the other hand, we have made the extracted Wikipedia dumps available, together with information on how to use them. The code repository is available at `https://gitlab.com/distration/dsi-nlp-publib`, which contains a link to the cloud folder storing the datasets.

We provide below a response to the received feedback and the explanation of all major changes introduced. To facilitate the revision process, we provide a copy of the manuscript annotated in red and blue color, respectively marking removed and added content.

We note that the manuscript has undergone significant changes; we believe such extensive additions were necessary to address the critical issues pointed out by the reviewers, reviewer 1 in particular. We now present the addressed issues point-by-point.

Reviewer 1 pointed out that presenting the paper as "multilingual" might be misleading, as it includes a small fraction of the overall thousands of languages in the world. As such, we have overhauled our work to put more emphasis on the fact that our aim was to gauge how well various classification methods could be adapted to a real-world scenario with limited resources, and that our perspective was mainly on the Italian language.

We also agree that the analysis of TC procedures was too shallow and not related enough to language-specific issues. In order to improve it, we have split it into three more organized sections. The **Preprocessing** section describes in more detail those preprocessing operations related to language, with a large focus on text segmentation. Indeed, this is the largest addition to the manuscript. The other two sections largely replace and expand the old "TC procedures" section. The **Text representation** section goes into more detail about how text is projected into feature space as we underline the importance of proper text representation in any NLP procedure, especially from a linguistic point of view. Lastly, we've added a briefer section which provides some insight on the **Classification step** of the pipeline.

We've clarified the meaning of the "computational resources" section as a severe bottleneck to experimentation with recent language models, which is not a linguistic issue *per-se* but it has direct impact on the possibility of evaluating these models. In fact, our limited resources were one of the reasons why we could not perform more accurate experimentation. We hope that, by improving the previous sections, as well as specifying this section in more detail, it is clearer that computational resource requirements bring daunting issues to the adoption of NLP methods in other languages.

Though we have slightly revised it, we find ourselves at a disagreement on the points brought about the analysis of datasets. Indeed, such research was meant to highlight the scarcity of downstream, task-specific datasets for the Italian language, which could only be proven by showing empirical data. Again, we disagree on the point that such datasets can be found with not too much effort, as can be proven by their scarce adoption in the literature. In our experience, many of these datasets are scattered around the web and cannot be reliably accessed through a single search tool. Moreover, we have found multiple references to existing datasets, only to find that they had been retired, made private or otherwise rendered unavailable. Nonetheless, we hope that this section can come across more smoothly after the revision done to the other parts of the manuscript.

We agree on the critiques related to the experimental section, and have added quite a few considerations to the analysis. We've added variance statistics in the performance results, as well as distribution of labels for all datasets. Details about initialization and optimization procedures were already present in the supplemental material provided, which has however been further expanded to explain the reason behind the choice of methods and to clarify further other technical details.

Lastly, Reviewer 2 has asked us to add some references with regards to the analyzed Reuters database, which we have done.

We confirm that neither the manuscript nor any parts of its content are currently under consideration or published in another journal. All authors have approved the manuscript and agree with its submission to *PLOS ONE*.

Sincerely yours,

Andrea Gasparetto (corresponding author)

andrea.gasparetto@unive.it

April 15, 2022
